# Atg8ylation as a general membrane stress and remodeling response

**DOI:** 10.15698/cst2021.09.255

**Published:** 2021-08-12

**Authors:** Suresh Kumar, Jingyue Jia, Vojo Deretic

**Affiliations:** 1 Autophagy Inflammation and Metabolism Center of Biomedical Research Excellence, University of New Mexico Health Sciences Center, Albuquerque, NM 87131, USA.; 2 Department of Molecular Genetics and Microbiology, University of New Mexico Health Sciences Center, Albuquerque, NM 87131, USA.

**Keywords:** ubiquitylation, endosome, exosomes, microvesicles, secretory autophagy, unconventional secretion, secretion, ubiquitin, galectin, lap, lc3, atg8, tfeb, lysosome, ampk, mtor, autophagy

## Abstract

The yeast Atg8 protein and its paralogs in mammals, mammalian Atg8s (mAtg8s), have been primarily appreciated for their participation in autophagy. However, lipidated mAtg8s, including the most frequently used autophagosomal membrane marker LC3B, are found on cellular membranes other than autophagosomes. Here we put forward a hypothesis that the lipidation of mAtg8s, termed ‘Atg8ylation', is a general membrane stress and remodeling response analogous to the role that ubiquitylation plays in tagging proteins. Ubiquitin and mAtg8s are related in sequence and structure, and the lipidation of mAtg8s occurs on its C-terminal glycine, akin to the C-terminal glycine of ubiquitin. Conceptually, we propose that mAtg8s and Atg8ylation are to membranes what ubiquitin and ubiquitylation are to proteins, and that, like ubiquitylation, Atg8ylation has a multitude of downstream effector outputs, one of which is autophagy.

## INTRODUCTION

The yeast Atg8 protein and its seven mammalian Atg8 paralogs (mAtg8s) that include LC3A, LC3B, LC3B2, LC3C, GABARAP, GABARAPL1, and GABARAPL2/GATE16 [1–3] are best known for their role in autophagy [4], a process described early on along with the definition of lysosomes [5, 6]. Canonical autophagy is a metabolic and cellular quality control process [7] which typically sequesters cytoplasmic cargo into double-membrane autophagosomes decorated with mAtg8s [8] and typically delivers the cargo to autolysosomes via fusion between autophagosomes and lysosomes [9]. The default termination of the canonical pathway in autolysosomes is degradation of the captured cytoplasmic material [8]. However, mAtg8s are found on a variety of other membranes in diverse biological and physiological contexts, including LC3-associated phagocytosis (LAP) and its variations [10]. Currently, many of these and related phenomena are grouped under the umbrella of ‘non-canonical autophagy' [10], although some of them such as LAP lack cytosolic cargo, which in principle defines the term ‘autophagy' or ‘self-eating'. As an alternative to ‘non-canonical autophagy', here we propose the ‘Atg8ylation hypothesis' as a principle that can unify different manifestations and roles of mAtg8s, their lipidation, and their association with various membranes. We propose that mAtg8ylation is a cellular response that both counters membrane stress and is a mechanism involved in general membrane remodeling, with canonical autophagy being one manifestation.

**Figure 1:**
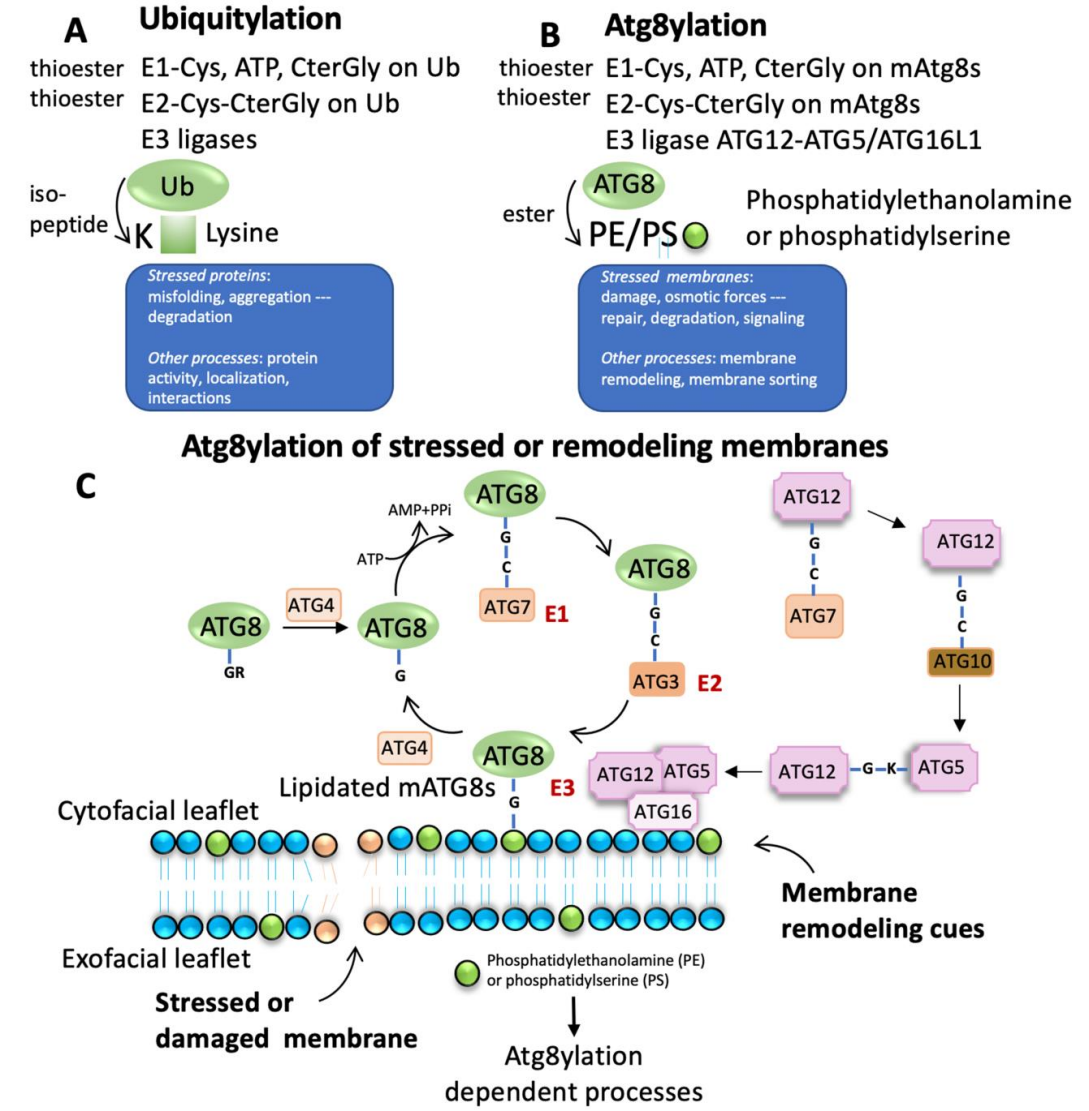
FIGURE 1: Comparison between ubiquitylation and Atg8ylation. **(A)** Principal components and steps of ubiquitylation. Note that misfolded or aggregated or otherwise engaged proteins are the principal targets for ubiquitylation. **(B)** Principal components and steps of Atg8ylation. Note that stressed membranes or membranes destined for a specific kind of remodeling are the principal targets for Atg8ylation. **(C)** The well-established lipidation cycle of mAtg8s (mammalian Atg8s: LC3A,B,C, GABARAP, GABRAPL1 and GABARAPL2) on target membranes. Green circles, polar groups of PE or PS.

**Figure 2:**
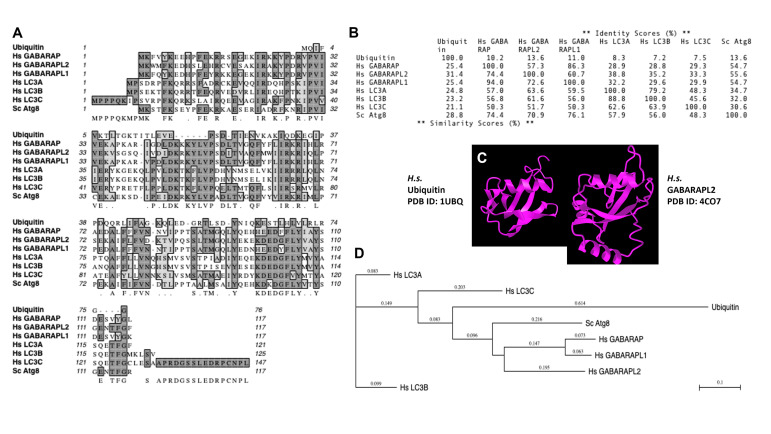
FIGURE 2: Similarities between ubiquitin and mAtg8s. **(A)** Multiple sequence alignment. **(B)** % similarities. **(C)** Crystal structure comparison between ubiquitin and GABARAPL2. **(D)** Evolutionary relatedness tree.

Of note, mAtg8s are related to ubiquitin in sequence and structure (**Figures 1** and **2**). Ubiquitylation is associated with disassembly, degradation and removal of misfolded proteins triggered by stressors [11–13]. Ubiquitin protein modifications [14] also play other non-degradative roles, such as modulation of normal protein activity, localization and interactions under unperturbed conditions [11–13]. We hypothesize that mAtg8s and Atg8ylation play a role in processes maintaining membrane homeostasis under stress conditions as well as in normal membrane remodeling in response to programmed, physiological or pathological cues. We propose here that Atg8ylation (**Figure 1**) is to membranes what ubiquitylation is to proteins.

## ATG8YLATION AND UBIQUITYLATION

Both autophagy [8], introduced above, and the ubiquitin-proteasomal system (UPS) [11, 12] are major modulatory machineries in eukaryotic cells which are often engaged in cargo degradation but are also performing multiple other functions [10, 13, 15]. Both systems require tagging of the cargo/target to be processed or acted upon.

Ubiquitylation of proteins often occurs on misfolded and aggregated proteins caused by stressors as well as under unperturbed conditions, modulating normal protein activity, localization, and their interactions (**Figure 1A**). Atg8ylation is a process whereby the Atg8 conjugation machinery is recruited to damaged or otherwise stressed membranes or to membranes undergoing remodeling under various homeostatic or non-homeostatic conditions (**Figure 1B**) and catalyzes mAtg8s' conjugation to phosphatidylethanolamine (PE) [16–18], or phosphatidylserine (PS) [18]. Like ubiquitylation, Atg8ylation involves an E1-like activating protein, ATG7 and requires ATP for activation (**Figure 1C**). ATG7 conjugates to the C-terminal glycine of mAtg8s, exposed post-translationally by ATG4 proteases. This is followed by action of the E2-like activity of ATG3 on ATG8s. Finally, the conjugation of mATG8s to PE or PS is mediated by an E3-like complex ATG5-ATG12/ATG16L1 (**Figure 1C**) [4, 19].

Of note, the diverse mammalian Atg8s are all related to ubiquitin (**Figure 2A-C**), underscoring the similarities between the two systems and the biochemical relatedness of ubiquitylation and Atg8ylation consistent with the parallels in the conjugation cascade. The two differ principally in the type of targets that ubiquitin or Atg8s are being conjugated to – in the case of ubiquitin to the stressed and degradation-bound or otherwise modified proteins, whereas in the case of mAtg8s, to the stressed or otherwise engaged membranes. The diverse events downstream or associated with Atg8ylation are individually described in subsections below.

## CANONICAL AUTOPHAGY

As mentioned above, mAtg8s and Atg8ylation have been initially almost exclusively associated with the process of autophagy [5, 6] controlled by an ensemble of Atg factors, first genetically defined in yeast [12, 20, 21] and conserved in mammals [8, 19]. Canonical autophagy has been appreciated primarily as a degradative process [4], with metabolic and quality control functions [7]. Whereas the degradative function of UPS involves elimination of individual proteins [22], autophagy can eliminate protein aggregates and larger structures, including whole organelles or their parts [23]. Autophagy is initiated in response to various cellular stressors, and requires the lipid kinase VPS34 (PIK3C3), first identified in yeast as a PI3P-generating enzyme in response to stress such as membrane-stretching osmotic changes [24]. The details of the autophagy pathway have been reveled through genetic analyses in yeast [4, 12, 20, 21] with a suite of ATG genes controlled by upstream kinases AMPK [25–27], mTOR (mechanistic target of rapamycin) [26, 28, 29] and TBK1 [30–34].

Conventionally, autophagy initiation is controlled by several modules [19]: (i) The ULK1/2 kinase complex with FIP200, ATG13 and ATG101, acting as conduits for inhibition by active mTOR [28, 29, 35] and activation by AMPK [25] to induce autophagy; (ii) ATG14L-endowed Class III PI3-Kinase Complex [36–38] that includes VPS34 and Beclin 1 [39], which can also be modified by AMPK to specifically activate the ATG14L form of VPS34 [40]; (iii) ATG9, and the ATG2-WIPI protein complexes [41–43]. These modules become interconnected, via FIP200 that bridges the ULK1/2 complex with the mAtg8s conjugation system by binding ATG16L1 [44–46], via ATG16L1 and WIPI interactions [47], and ATG13 connecting the ULK1/2 complex with ATG14-VPS34 [48, 49]. After initiation, autophagy terminates in a merger of autophagosomes with degradative endolysosomal compartments whereby the sequestered cargo is degraded. This is catalyzed by a suite of SNAREs, e.g. STX17 [50] YKT6 [51], VAMP8, and SNAP29 [50].

Canonical autophagy and its variations manifested in different forms of selective or quality control autophagy, defined by the captured cargo (mitophagy, ER-phagy, aggrephagy, xenophagy, ribophagy, pexophagy, lipophagy, and precision autophagy of individual protein complexes) [7, 8] depend on the above apparatus to be guided by the receptors and receptor-regulators, many of whom belong to the category termed Sequestosome-like receptors (SLRs) named after the founding member Sequestosome 1 (SQSTM1/p62) [52]. The majority of autophagic receptors interact with mAtg8s, albeit interaction directly with FIP200 is another “entry” into the pathway [33, 34, 53, 54], likely amplified by eventual mAtg8 binding [53]. To interact with mAtg8s, SLRs contain dedicated Atg8 interacting motifs (AIM), also known as LIRs (LC3 interacting regions). As an interesting crossover to the ubiquitin system, SLRs often recognize ubiquitylated cargo via dedicated ubiquitin binding domains [55]. Thus, Atg8ylation and ubiquitylation act here as coincidence detectors for efficient cargo degradation.

**Figure 3:**
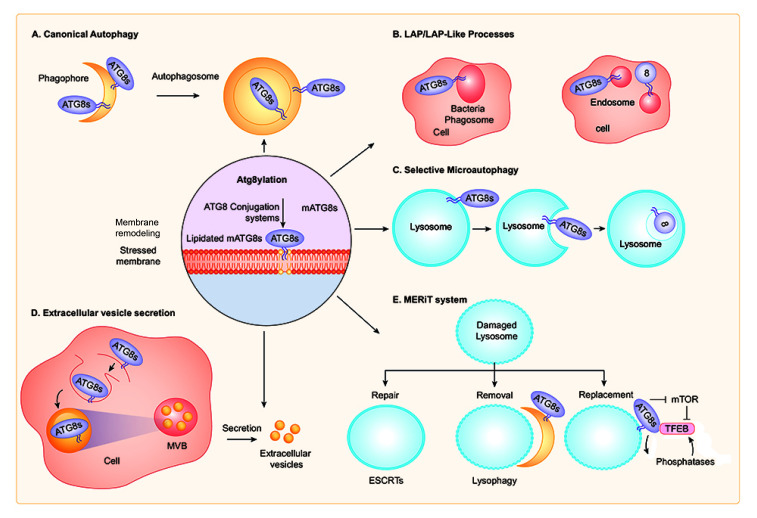
FIGURE 3: Different forms and roles of Atg8ylation. **(A)** Canonical autophagy. Note double membranes, as phagophores close around the autophagic cargo, and presence of lipidated mAtg8s on the outside and inside of the double membrane autophagosome, however, always on the originally cytofacial leaflet. This is the well-established macroautophagy pathway. **(B)** LAP and LAP-like processes (Lapoid) occurs on phagosomes and endosomes whose cytofacial leaflet of (single membrane) delimiting the organelles is Atg8ylated upon ecountering physical stress signals. **(C)** Selective microautophagy occurs via an MVB generation-like process whereby lysosomal delimiting membrane that is Atg8ylated invaginates and reduces the surface, size, and contents of the lysosomes. **(D)** Extracellular vesicle secretion is a subset of secretory autophagy, and in principle represents similar topological changes (albeit differing in details and regulatory processes involved) as in selective microautophagy, except that the cargo sequestered into the MVB-like bodies is secreted upon exocytosis instead of being degraded. Note that EV secretion is a component of a broader collection of secretory autophagy modalities (not shown). **(E)** MERiT system involves a coordinated response to lysosomal damage, with ESCRTs conducting the repair of mildly damaged lysosomes, autophagy (‘lysophagy') removing extensively damaged lysosomes, and TFEB initiating a lysosomal replenishment program. Note that Atg8ylation thus far has been demonstrated to play a role in the lysophagy and TFEB-dependent steps. See details in the text for each type of Atg8ylation's manifestations including mechanisms and physiological roles. All events are intracellular, even when cells are not depicted.

Autophagosomal membrane formation is believed to entail mAtg8 conjugation machinery and Atg8ylation, i.e. Atg8 lipidation on membranes that form autophagosomes (**Figure 3A**) [19]. However, recent studies indicate that mAtg8s may not be absolutely required to form autophagosomal membranes as their absence mostly have limited kinetic or autophagic vesicle size effects [56, 57]. This is despite mAtg8's iconic status, since LC3B is used as an almost quintessential marker of autophagosomes [58]. Atg8ylation may play additional roles in autophagy, for example as a modulator of the recruitment and/or function of SNAREs. This includes a large subset of AIM (LIR)-containing SNAREs including STX3, STX4, STX6, STX16, STX17, STX19, Vti1a, GOSR1, and VAMP7 [59–61], several of which are important in control of general membrane fusion and flow, as well as specifically for autophagosomal [32], autolysosome [50], and lysosomal [61] biogenesis. There are indications that Atg8ylation plays a role in modulating these SNARE-controlling mAtg8 activities, since expression of a dominant negative ATG4 that prevents lipidation of all mAtg8s inhibited STX17 function [60] in the autophagosomal pathway.

## LAP AND LAP-RELATED PROCESSES

LAP [10, 62], LANDO (LC3-associated endocytosis) [63], and a cluster of other related phenomena involving phagosomes or stressed endosomes [64–68] to which we refer collectively here as Lapoid processes, represent a form of Atg8ylation occurring on single membranes of endosomes and phagosomes (**Figure 3B**). LAP per se lacks cytosolic cargo, but some Lapoid processes involve cytosolic cargo. LAP involves Atg8ylation, specifically of LC3 subfamily of mAtg8s [69], on membranes of phagosomes taking up stress- or signal-inducing cargo such as potentially dangerous pathogens, inflammation-inducing dead cells, live cells to be eliminated by entosis, and extracellular debris or aggregates [62, 65, 68–70]. Of note, not every endosome and phagosome undergoes Atg8ylation, and it takes a specific stress or danger signal such as presence of TLR (Toll-like receptor) ligands within the phagocytosed or endocytosed material [62]. TLR signaling recognizes fungal, bacterial and microbial products commonly known as PAMPs (pathogen associated molecular patterns), and in principle induces strong Atg8ylation of endomembranes [71].

The key distinguishing feature of LAP, LANDO, and other Lapoid processes that differentiates them from canonical autophagy is the absence of double membranes, i.e., Atg8ylation occurs on the delimiting single bilayer membrane of the stressed endosomes and phagosomes. Further key differences are the dependence of LAP on RUBCN (Rubicon), which is an inhibitor of canonical autophagy, and its independence of FIP200, which is required for canonical autophagy and Atg8ylation of conventional double membrane autophagosomes.

In principle, a similar mAtg8 lipidation machinery is involved in Lapoid phenomena as in the case of canonical autophagy. It is the ATG16L1 component of the Atg8ylation E3 ligase (ATG5-ATG12/ATG16L1) that discerns between target membranes that are to be Atg8ylated [17]. Lipid binding domains are found in ATG16L1 that govern canonical autophagy vs. Lapoid phenomena [17]. The C-terminal membrane-binding region of ATG16L1 is dispensable for canonical autophagy but important for Lapoid processes [17]. The N-terminal membrane-binding amphipathic helix of ATG16L1 is required for Atg8ylation (specifically demonstrated in LC3B lipidation assays) in both canonical autophagy and Lapoid phenomena [17].

The upstream machinery that recognizes stressed membranes remains to be elucidated, but may involve signals form TLRs, stretching of lipid bilayers during osmotic stress, ion transport events, etc. One upstream signal that has been clearly established in LAP is NOX2 (NADPH oxidase) [72], which is classically activated upon opsonized-pathogen uptake by phagocytic cells to generate reactive oxygen species (ROS) [73]. Whereas it is not completely clear how NOX2 or NOX-generated ROS function to promote LAP, ROS can inhibit ATG4, an enzyme catalyzing mAtg8 dilapidation reaction (equivalent to DUB activity on ubiquitinated proteins) [74]. This could locally, i.e., in the vicinity of a phagosome undergoing LAP, stabilize lipidated mAtg8s.

How do LAP or Lapoid processes terminate, is another open question. Phagosomes that are Atg8ylated (LAP) appear to fuse with lysosomes in a more efficient way [62]. The mechanism of how mAtg8s increase fusion of LAPosomes may be similar to that of how autophagosomes fuse with lysosomes. It is possible that Atg8ylation in the case of LAP stimulates an equivalent process as in the case of Atg8ylation recruiting/activating STX17 on autophagosomes via AIM/LIR motifs [60].

LAP and Lapoid functions include anti-inflammatory processes such as those failing in lupus [75], elimination of pathogens [72, 76], removal of dead cells and efferocytosis [77, 78], entosis [65], and removal of rod outer segments via phagocytosis by retinal pigment epithelium cells [79]. LAP may promote cancer progression by favoring immune-tolerance in the cancer microenvironment, whereas defects in LAP elicit pro-inflammatory cytokines in tumor-associated macrophages [80]. LAP, however, can be pro-inflammatory by promoting TLR signaling through IRF7, which leads to type I interferon response [81]. LANDO deficient microglia display hyper-neuroinflammation and neurodegeneration; LANDO may be important for clearance of β-amyloid aggregates and suppression of microglia activation [63]. Physiological functions of many other Lapoid processes are yet to be defined.

## SELECTIVE MICROAUTOPHAGY

Selective microautophagy of mammalian lysosomal membranes (**Figure 3C**) and its proteins occurs in response to osmotic stress or glucose-starvation, adjusting the size of lysosomes under stress conditions [82]. This requires lipidation of mAtg8 and indicates that Atg8ylation participates in microautophagy [82]. It is unaffected by inactivation of ATG13, a member of the ULK1-FIP200-ATG13-ATG101 complex, akin to other Lapoid activities. This is another example of the mATG8s lipidation machinery being recruited to single bilayer endolysosomal membranes and serves here specifically to downsize lysosomes or control their surface-to-volume characteristics under stress conditions.

It is unclear how other microautophagy processes differ from the selective microautophagy described above. In principle, microautophagy represents a collection of processes, best characterized in yeast, which involve direct internalization of cytoplasmic cargo into the lumen of lysosomes by invaginations of the delimiting single bilayer lysosomal membrane [83–86]. Yeast microautophagy requires the action of most Atg proteins [86], and may be different from microautophagy (endosomal, multivesicular bodies, and selective microautophagy) in mammalian cells. The selective microautophagy phenomena in principle engage components of the ATG conjugation machinery [82], ESCRT machinery [87, 88], and a subset (but not all) SLRs [88], and may in some instances depend on Atg8ylation. Starvation-induced degradation of SLRs, other selective autophagy receptors, and ferritinophagy receptor NCO4 is independent of mTOR, which negatively controls canonical autophagy, and, consistently with that, depletion of FIP200, an essential component of canonical autophagy that transduces mTOR signals [89], does not prevent starvation-induced degradation of certain SLRs and NCAO4 [88]. Nevertheless, depletion of ATG7 blocks (at least in part) degradation of certain SLRs, specifically p62 and NDP52 [88], this strongly suggests that this process likely involves Atg8ylation on respective endolysosomal membranes. It is not known whether and to what extent Atg8ylation affects classical mammalian endosomal microautophagy, e.g. multivesicular bodies.

In another example of the role of Atg8ylation in selective microautophagy, excess ER during recovery from ER stress undergoes piecemeal micro-ER-phagy and requires mAtg8 lipidation [87].

## UNCONVENTIONAL SECRETION: EXTRACELLULAR VESICLES AND SECRETORY AUTOPHAGY

The emerging role of Atg8ylation in generating extracellular vesicles (EVs) mirrors its role in selective microautophagy. Microautophagy directly carried by lysosomes and endosomes can in principle result in sequestration, degradation or secretion of intralumenal vesicles (ILVs). The exocytosed ILVs in the extracellular space are referred to as exosomes, a subtype of EVs [90]. EVs are a collection of heterogeneous membranous structures coming in variety of forms and ranging from submicron to several microns in size and include microvesicles (also known as ectosomes) and exosomes [91]. Microvesicles directly bud off the plasma membrane after its outwardly evagination, whereas exosomes originate from endomembranous ILVs, both, however, undergoing the same, exofacial direction of budding [92]. EVs are present in biological fluids with a multitude of physiological effects [92], but are yet to be fully defined in terms of specificity of cargo packaging and associated functions [93]. EVs were originally identified as vehicles for selective elimination of proteins, lipids and RNA from cells [92, 94], however, EVs have now emerged as a means of intercellular communication [92, 95, 96].

Atg8ylation participates in the formation of exosomes [97, 98] and secretion of specific cytosolic cargo by EVs [99] (**Figure 3D**). Proteomic and RNA profiling of EVs identified diverse RNA binding proteins and small non-coding RNAs requiring LC3 and the Atg8ylation machinery for packaging and secretion [99]. Among other potential functions reported for exosomes impacted by Atg8ylation, one is the decoy sequestration of bacterial membrane damaging toxins before they can reach and attack the cellular plasma membrane [100].

Atg8ylation also plays a role in the type of unconventional secretion or excretion/extrusion of cytoplasmic material referred to as secretory autophagy [101, 102], with cargo ranging from individual proteins to intracellular organelles or pathogenic microorganisms [101], such as mycobacteria [103] and specific viruses [104]. Atg8ylation furthermore promotes certain types of viral budding at the plasma membrane, as in the case of filamentous mode of budding of influenza A [105]. The prototypical cytokine cargo for secretory autophagy are IL-1β [106–109], which does not have a leader peptide and thus is synthesized as a cytosolic protein [110], and additional cytokines such as IL-6 [111] and alarmins such as HMGB1 [107, 112]. However, IL-1β release from cells utilizes multiple routes. Such alternative pathways include import of the cytosolic IL-1β into the lumen of ERGIC, an intermediate compartment within the canonical secretory pathway [113, 114]. The most dominant route of passive release of IL-1β from dying cells is through gasdermin pores on the plasma membrane during pyroptosis [115], and IL-1β leaks through such pores even before the ‘official' cell death [116]. Whether and how Atg8ylation interfaces with various forms of unconventional secretion [117] remains to be fully explored and associated mechanisms understood.

## ATG8YLATION AS A RESPONSE TO MEMBRANE STRESS AND DAMAGE

Biological membranes provide a key diffusional barrier and define the physical boundaries of cells and their intracellular compartments; they are endowed with selective permeabilities, channels, pumps, and signaling systems [118, 119]. A number of stress conditions affect integrity and function of cellular membranes [120], which are in principle two-dimensional liquids of hydrated lipid bilayers composed of phospholipids, sterols, sphingolipids, integral and peripheral membrane proteins often decorated by glycocalyx [119]. To counter inherent membrane fragility, all cells have mechanisms to counter and repair damage [120], with morphologically visible measures taken by microbes, fungi and plants that stabilize their cells by rigid cell walls countering the osmotic stress among other environmental insults. Physical membrane damage [120], programmed or unprogrammed permeabilization transitions [115, 121, 122], osmotic stress [123], lipid tension or fluidity changes due to composition or temperature changes, exposure to microbial and environmental toxins and detergent-like molecules, as well as entropic changes, if not countered by continuous repair/replenishment functions [120], can perturb membranes either functionally or physically, with manifestations in pathological states and disease [118].

The plasma membrane is by its boundary nature under continuous risk of being damaged by exogenous agents, and conversely is used for programmed permeabilization events associated with cell death pathways. Recent studies [124–126] suggest that Atg8ylation-dependent processes and other ATG proteins engaged in non-canonical functions contribute to the protection of plasma membrane against physical damage (modeled by laser damage or detergent treatment), programmed permeabilization by gasdermin-pores during pyroptosis or MLKL during necroptosis, by MLKL-like permeabilization function of SARS-CoV-2 ORF3a, bacterial pore forming toxins (pneumolysin, lysteriolysin and streptolysin O), and harsh components of the cell walls of microbes such as *Mycobacterium tuberculosis* [124, 126]. Whereas ATG9A engages ESCRT proteins to seal plasma membrane holes immediately upon permeabilization [126], Atg8ylation results in blebbing due to lysosomal exocytosis [124] or endocytic processes termed LC3-associated micropinocytosis which appears to be a Lapoid process [125].

Intracellular membranes can also come under the physical assault or be subjected to membrane stress, such as osmotic changes. The lysosome is the digestive organelle of the cell and thus receives cargo destined for degradation from both cellular interior and the exterior space, via autophagic, endosomal and phagocytic pathways [127]. By the very nature of receiving various material from different tributary pathways, lysosomal membranes and organelles of the endolysosomal system are at risk of being damaged by the ingested material [128], lysosomotropic compounds [129], osmolytes such as trehalose [130], neurotoxic protein aggregates including α-synuclein, tau, and huntingtin amyloids [131–133], mineral crystals such as silica, monosodium urate, and calcium phosphate [134–136], pathogens, PAMPs and DAMPs (danger associated molecular patterns) [137, 138], and can be influenced by programmed events such as TRAIL (tumor necrosis factor-related apoptosis-inducing ligand) and downstream signaling [122, 131, 139–142].

Lysosomal damage elicits a set of diverse responses to restore lysosomal homeostasis [132, 143–148], reinstate key functions including delivery of iron [149], and adjust cellular metabolic needs by suppressing mTOR and stimulating AMPK while simultaneously mobilizing homeostatic responses requiring mTOR inhibition and AMPK activation [142, 150]. Early dominant events include responses by galectins [133, 138, 143, 151, 152], ubiquitin [132, 137, 142, 144, 145], ESCRTs [133, 146, 147], and include additional events [148] including recruitment of mAtg8s [153], which engage not only in canonical autophagy [46, 135] but also in other Atg8ylation-dependent processes [154, 155] elaborated in the subsequent section. Eventually, if membrane repair fails, a type of canonical autophagy termed lysophagy [46, 133, 135, 143, 145, 150] eliminates excessively damaged lysosomes, endosomes or phagosomes [138].

## ATG8YLATION SIGNALING AND TFEB RESPONSE IN LYSOSOMAL BIOGENESIS

Atg8ylation contributes to signaling events that activate TFEB (transcription factor EB) to initiate the lysosomal protein expression program. Starvation and additional signals, e.g., lysosomal damage, induce TFEB translocation form the cytosol to the nucleus where it stimulates lysosomal gene expression, and to an extent several autophagy genes [156–158]. Apart from its role in lysosomal biogenesis, TFEB transcriptionally controls other biological processes [127] including lipid catabolism [159], lysosomal exocytosis [160], mitochondrial biogenesis [161] and mitophagy [162]. TFEB activity is controlled by mTOR [127, 163, 164]. TFEB phosphorylation by mTOR increases its binding to 14-3-3 proteins, which holds TFEB in the cytosol [163, 165]. TFEB is under a special control by mTOR, downstream of the inputs from Foliculin/FLCN to RagC/D, in contrast to other signals transduced to mTOR via RagA/B [166]. Protein phosphatase 3 catalytic subunit beta (PPP3CB) dephosphorylates TFEB and releases it from 14-3-3 [165]. Additional studies reporting calcium-dependent but PPP3CB-independent dephosphorylation of TFEB indicate possible involvement of another phosphatase [155].

TFEB is activated by a variety of membrane and other stress conditions including starvation [154, 164], lysosomal damage [133, 143, 155], pathogen infections [154, 167–170] and mitochondrial stress [162]. Atg8ylation controls nuclear translocation of TFEB [154, 155, 171] (**Figure 3E**). TFEB interacts directly with GABARAP [154] and the GABARAP subset of mAtg8s supports nuclear translocation and transcriptional activity of TFEB during starvation [154] and lysosomal damage [155]. Calcium flux stimulates nuclear translocation of TFEB [155, 165]. Atg8ylation affects intracellular calcium levels [154], whereas transcription of the lysosomal calcium channel TRPML1 [165] is reduced in mAtg8 depleted cells [154]. Furthermore, TRPML1 interacts with lipidated LC3 in response to lysosomal damage [155], which is a clear example of Atg8ylation controlling molecular events other than lysophagy.

Atg8ylation has appreciable effects on TFEB activity during starvation, lysosomal damage of xenophagy [154, 162], [155, 171]. GABARAP binds FLCN/FNIP complex [171], which is a GAP (GTPase activating protein) for RagC/D GTPase, and activates (paradoxically) mTOR when amino acids are aplenty [172, 173]. However, under membrane stress conditions, binding of FLCN to membranes Atg8ylated by GABARAP disrupts the regulation of RagC/D by FLCN, impairing TFEB phosphorylation by mTOR and activating TFEB [171]. In conclusion, Atg8ylation has significant and multiple roles to in control of TFEB activity.

## CODA

The herein proposed unifying concept of Atg8ylation refers to a process of tagging stressed membranes with lipidated mAtg8s. This includes damaged, or otherwise mobilized and remodeling membranes leading to a plethora of outcomes. Often, these processes occur on endosomal, lysosomal and autophagosomal membranes but may not be limited to these organelles. The spectrum of presentations includes canonical autophagy, LAP and Lapoid processes, selective microautophagy and unconventional secretion, different stages of the MERiT lysosomal QC system, and membrane modification during programmed or signaling events.

The elucidation of the canonical autophagy pathway in yeast has provided a clear linear pathway of Atg8-driven steps, from autophagosome formation to their fusion with the vacuole/lysosome. In mammalian systems, this pathway and its core components are conserved. The presence of multiple Atg8 paralogs in mammals reflects increased complexity, whereas experimental analyses have revealed that mAtg8s function in processes other than autophagy. The proliferation of these ‘non-canonical autophagy-related' processes may at first appear confusing. The Atg8ylation concept proposed here resets the view of what lipidation of mAtg8s represents considering its standalone manifestation and nature – it is a covalent modification of membranes the way ubiquitin covalently modifies proteins. Once this point of view is considered, it is relatively easy to understand how this can participate in a multitude of processes, of which canonical autophagy is just a subset. This could be useful as more and more ‘anomalies' or non-canonical processes are being described, and should spur further investigations based on the principle of Atg8tylation freed from the conceptual confines, however elegant, of the canonical autophagy pathway. Retrospectively, membrane processes such as LAP, LANDO, selective microautophagy, EV/exosome secretion, etc., can be easier to understand from this point of view, while stimulating future systematic discoveries.

The crossovers between ubiquitylation and Atg8ylation exist, as glimpsed from emerging examples of ubiquitylation being able to occur on complex glycolipids such as bacterial LPS (lipopolysaccharide) when introduced into the mammalian cell [174], potentially blurring the borders of specialization between ubiquitylation and Atg8ylation relative to proteins vs. membranes. Conversely, two recent reports of protein Atg8ylation [175, 176] confirm that Atg8ylation and ubiquitylation are more similar than previously appreciated, although the biological significance of protein Atg8ylation is yet to be explored. In addition to proposing the concept of Atg8ylation, as the membrane modification equivalent to the ubiquitylation of proteins, this review collates a number of seemingly disparate phenomena into a unified model. The concept of Atg8ylation offers a new perspective on the system usually associated with autophagy and may help in developing approaches to pharmacologically manipulate Atg8ylation rather than the entire canonical autophagy pathway.

With the introduction of Atg8ylation as a concept some old questions remain, and new questions arise: What exactly are the functional and mechanistic consequences of Atg8ylation in general and in specific examples covered here and how and does the lipidation of mAtg8s affect their functionalities? Do Atg8ylated membranes share specific types of lipid modifications, changes in ordering/tension within the bilayer, or other molecular features that are specific to ‘stress' or a ‘stress' signal? When a target protein reaches a threshold of ubiquitin modifications this often leads to its proteasomal degradation; could Atg8ylation similarly operate in the context of membrane remodeling and degradation? Doe Atg8ylation also take place under basal conditions, and, if so, would there be quantitative and qualitative differences between basal vs. ‘stress' conditions? With Atg8ylation expanding the functional roles of mAtg8s to outside of the cells through unconventional secretion, do these processes serve to excrete material from the cells or take active signaling roles such as in the case of EVs in intercellular communication? What are the specific roles of mAtg8s and Atg8ylation in processes of unconventional/secretory autophagy, and do they play a role in cargo selection or vesicle formation or both?

As the most celebrated Atg8ylation process, canonical autophagy impacts a spectrum of pathophysiological conditions including cancer, neurodegenerative diseases, metabolic and cardiovascular disorders, autoimmunity, inflammation and infection [4, 177, 178]. LAP and Lapoid processes are also physiologically important [10], whereas other Atg8ylation processes are slowly establishing biological and potential medical relevance. The broader view presented here of membrane-specific (albeit not exclusive) modification via Atg8ylation should open both fundamental and translational approaches different than those based on autophagy alone.

## Abbreviatons:

AIM: – Atg8-interacting motif;
EV: – extracellular vesicle;
ILV: – intralumenal vesicle;
LANDO: – LC3-associated endocytosis;
LAP: – LC3-associated phagocytosis;
LIR: – LC3 interacting region;
mAtg8s: – mammalian Atg8s;
mTOR: – mechanistic target of rapamycin;
PAMP: – pathogen associated molecular pattern;
PE: – phosphatidylethanolamine;
PS: – phosphatidylserine;
ROS: – reactive oxygen species;
SLR: – sequestosome-like receptor;
TFEB: – transcription factor EB;
TLR: – Toll-like receptor;
UPS: – ubiquitin proteasome system.
